# Errata in Last Number

**Published:** 1821-10

**Authors:** 


THE LONDON

### Medical and Physical Journal.

4 OF VOL. XLVI.] OCTOBER, 1821. [NO. 272.

NOTICE.

*Another of the series of* PROEMIA *to the several volumes of this Journal, (which commenced with that to the forty-third volume,) comprising a History of the Progress of Medicine and its auxiliary Sciences for the half-year immediately previous to the period of their production, respectively, was published on the last day of July. One of the especial intentions of those* Pröemia, *is to present a comprehensive view of the state and progress of Medicine throughout Europe generally, and in the United States of America; an object that cannot be effected in the regular monthly Numbers of the Journal, because of the small extent of space which can be there appropriated to this purpose.*

### MONTHLY CATALOGUE OF MEDICAL BOOKS.

Lectures on the Structure and Physiology of the Male Urinary and Genital Organs of the Human Body, and on the Nature and Treatment of their Diseases. By James Wilson, F.R.S. 8vo. 14s.

Essays on the Female Economy. By John Power, M.D. 8vo. 4s. 6d.

A Manual for the Student of Anatomy; containing Rules for displaying the Structure of the Body, so as to exhibit the Elementary Views of Anatomy, and their Application to Pathology and Surgery. By John Shaw. 12mo. 10s.

Outlines of Midwifery, developing its Principles and Practice; intended as a Text-book for Students, and a Book of Reference for junior Practitioners. By J. T. Conquest, M.D. F.L.S. Second edition, enlarged. 12mo. 7s. 6d.

*THE Readers of the London Medical and Physical Journal will have perceived, from the indication on the cover of the present Number, that Dr. Hutchinson has ceased to be the conductor of that Work.*

*An appointment, which will shortly call Dr. Hutchinson to Russia, has caused him to relinquish the Editorship; and, though he believes that the readers of the Journal will have ample occasion for gratification from the change, he is not willing to appear insensible of his obligations to those who have patronized the Journal whilst it continued under his superintendance, by neglecting to offer them his most grateful thanks, and by not expressing his acknowledgments of their favours to those especially who, by placing at his disposal the many estimable papers which, during the last three years, have been inserted in the department for original communications, have contributed, in an essential degree, to the success of his efforts to render the Journal worthy of the attention of the Medical Profession.*

*London; Sackville-street, October* 1, 1821.

---

### NOTICES TO CORRESPONDENTS.

Mr. PURTON *and* Mr. C. C. WALLIS'S *Papers have been received, and are under consideration. We shall always be glad to hear from the former on subjects of importance connected with midwifery, which he states to have practised extensively.*

Mr. MARLEY'S *case of* angina pectoris *will be inserted in our next Number.*

*We regret that* Mr. ENGLISH'S *notions and our own respecting the importance of his communication do not coincide. We have, however, mentioned the* fact *it contained in his letter in another part of the Journal. The* Postscript *is an Advertisement.*

*A correspondent is informed that the* Essay on the Sensibility of Animals, *being the first of a series, is come safe to hand.*

*We rejoice at* Mr. JEFFERY DENNIS'S *recovery from tic-douloureux, by means of carbonate of iron. His communication partakes too much of the nature of a circular to be admitted in our Journal. Mr. J. D. must be aware that his case has already received the notoriety it deserves, for it has appeared in the Gazette of Health.*

Dr. ROBERT HAMILTON *will see from the readiness with which we have given insertion to his communication, how much we applaud the idea of our brethren beyond the Tweed. We shall always feel most happy in promulgating an account of the progress of the infant Medico-chirurgical Society of Edinburgh.*

*We trust that* Mr. JOSEPH HUME, *of Long-Acre, will soon be able to favour us with the case of which we have given an abstract outline in our Medical and Physical Intelligence. From our knowledge of his skill in practical chemistry, and of his having practised many years as an apothecary, we doubt not but his promised paper will be worthy of his reputation.*

Mr. TRIPE *has our best thanks for his valuable Essay, inserted in the present Number. His recent case of lithotomy will be read with much interest. We hope to hear from him again.*

*A. Z. is mistaken: we have never had, nor wish to have, any thing in common with a certain Journal.*

*We neither can nor feel inclined to admit the ribaldry against Sir W. Adams.* CASTIGATOR *has mistaken his man. He must look out for some other Journal, in which to vent his spleen. We admit, that four thousand pound and a high encomium, bestowed by a Committee of the British House of Commons, on a rival oculist, are rather "a sore eye."*

*We have long been aware of the fact which* PERMISSUS *wishes us to divulge; but we do not set up as reformers of irregularities, nor as the chastisers of quacks. True it is that the physician-accoucheur he alludes to belongs neither to the College of Physicians, nor to the College of Surgeons, nor to the Company of Apothecaries, and that he nevertheless practises in all the branches of our profession, which no one knows when or where he has learned; but* PERMISSUS *cannot, surely, be serious, when he expresses a desire to see us taking up a cause privately, which it is the duty of much higher authorities to defend on public grounds. We have no wish to make* men merry *at our own expense.*

*The Editor of the Medical and Physical Journal would fain flatter himself, that in his endeavours to support the reputation of the Journal he has undertaken to conduct, his personal friends, as well as the numerous friends of science, generally, will assist him, if not in increasing, at least in maintaining that degree of respectability for which this useful publication is indebted to the exertion of his worthy predecessor.*

