# Errata in Last Number

**Published:** 1821-11

**Authors:** 


					In oi.r Notice to Correspondents the compositor has inserted if, by mistake, line C, from the top ?
Slid, by uu unlucky transposition, in tUc huny of business, <m "eyesore" uas made " a sore-cyc."'

				

